# Host Range, Morphology and Sequence Analysis of Ten Temperate Phages Isolated from Pathogenic *Yersinia enterocolitica* Strains

**DOI:** 10.3390/ijms23126779

**Published:** 2022-06-17

**Authors:** Jens Andre Hammerl, Sabrin El-Mustapha, Michelle Bölcke, Hannah Trampert, Andrea Barac, Claudia Jäckel, Ashish K. Gadicherla, Stefan Hertwig

**Affiliations:** Department of Biological Safety, German Federal Institute for Risk Assessment, Max-Dohrn Str. 8-10, D-10589 Berlin, Germany; jens-andre.hammerl@bfr.bund.de (J.A.H.); sabrin-elmustapha95@outlook.de (S.E.-M.); michelleboelcke@web.de (M.B.); hannah.trampert@web.de (H.T.); andrea.barac@bfr.bund.de (A.B.); claudia.jaeckel@bfr.bund.de (C.J.); ashish.gadicherla@bfr.bund.de (A.K.G.)

**Keywords:** *Yersinia enterocolitica*, temperate phage, genome, attachment site

## Abstract

*Yersinia enterocolitica* is a heterogeneous species comprising highly pathogenic, weakly pathogenic and non-pathogenic strains. Previous data suggest that gene exchange may occur in *Yersinia*. Only scarce information exists about temperate phages of *Y. enterocolitica,* even though many prophage sequences are present in this species. We have examined 102 pathogenic *Y. enterocolitica* strains for the presence of inducible prophages by mitomycin C treatment. Ten phages were isolated from nine strains belonging to the bio (B)/serotypes (O) B2/O:5,27, B2/O:9 and 1B/O:8. All phages are myoviruses showing lytic activity only at room temperature. Whole-genome sequencing of the phage genomes revealed that they belong to three groups, which, however, are not closely related to known phages. Group 1 is composed of five phages (type phage: vB_YenM_06.16.1) with genome sizes of 43.8 to 44.9 kb, whereas the four group 2 phages (type phage: vB_YenM_06.16.2) possess smaller genomes of 29.5 to 33.2 kb. Group 3 contains only one phage (vB_YenM_42.18) whose genome has a size of 36.5 kb, which is moderately similar to group 2. The host range of the phages differed significantly. While group 1 phages almost exclusively lysed strains of B2/O:5,27, phages of group 2 and 3 were additionally able to lyse B4/O:3, and some of them even B2/O:9 and 1B/O:8 strains.

## 1. Introduction

*Yersinia enterocolitica* is an enteropathogenic species causing a gastrointestinal disease termed yersiniosis [1]. Yersiniosis was the third most commonly reported foodborne zoonotic disease in the European Union (EU) in 2019 [2]. In many countries, the presence of *Y. enterocolitica* is clearly associated with pigs [3,4] and the consumption of raw or insufficiently cooked pork is assumed to be the main cause of infection [5]. *Yersinia enterocolitica* is a heterogeneous species containing six biotypes (B: B1A, B1B, B2, B3, B4 and B5) and more than 70 serotypes (O) [6], not all of which are pathogenic. In Europe, most infections are caused by B4/O:3, B2/O:9, B2/O:5,27 and, to a lesser extent, by the highly pathogenic 1B/O:8 [7]. By contrast, B1A strains, which frequently occur in the environment, in food and also in pigs, are generally regarded as nonpathogenic. However, even though these strains mostly lack important virulence factors encoded by the chromosome (e.g., attachment-invasion locus, invasin) or by the virulence plasmid pYV (*Yersinia* adhesin, *Yersinia* outer proteins) only existing in the other five biotypes, their role in pathogenicity has to be clarified, because an increasing number of publications report on the isolation of B1A strains from clinical samples [8,9]. In this context, the question arises, whether there is gene transfer between pathogenic and nonpathogenic *Yersinia* strains. A conjugative *Y. enterocolitica* plasmid able to mobilize pYV has already been described [10,11,12]. Transduction might also be important for gene transfer in *Y. enterocolitica* because about 75% of isolates of this species were reported to be lysogenic [13,14]. However, only limited information is available on active prophages in *Yersinia*. A filamentous phage (YpfF) from *Y. pestis* has been studied, which contributes to the pathogenicity of this agent by enhancing the capacity of the plague bacillus to multiply and disseminate in mice [15,16,17]. Two studies have been published on the isolation of temperate phages from *Y. enterocolitica.* Popp et al. (2000) treated 170 *Yersinia* strains belonging to several species with mitomycin C and could induce eight prophages, six in non-pathogenic *Y. enterocolitica* B1A strains, one (PY20) in a pathogenic B4/O:3 strain and one in *Y. frederiksenii* [18]. The temperate phages were myoviruses (PY20, PY44, PY68, PY95), podoviruses (PY31, PY96) and siphoviruses (PY30, PY54) and showed a narrow to moderate wide host range. Phage PY20 was used for transduction experiments and able to transduce small *Y. enterocolitica* plasmids [19]. Moreover, the transductants were lysogenic and harboured the phage in their genome. Liang et al. (2019) isolated three very similar (99.99% identical) myoviruses (YeP1, YeP2, YeP3) from B3/O:3 strains, two podoviruses (YeP5, YeP6) from B2/O:9 and one podovirus (YeP4) from a B3/O:9 strain, which are closely related (99.9% identical with one another) [20]. The phages showed some similarity to phage PY54, whose prophage replicates in *Y. enterocolitica* O:5 and O:5,27 strains as linear plasmids with covalently closed ends [21,22]. Regrettably, Liang et al. (2019) did not report on the host specificity of their phages [20].

In this work, we analysed more than 100 pathogenic *Y. enterocolitica* strains for the presence of inducible prophages. We could isolate ten temperate phages, which were characterized regarding their morphology, host range and genome sequences and show that they belong to three novel groups of myoviruses.

## 2. Results

### 2.1. Nine Y. enterocolitica Strains Released Phages That Are Lytic at Room Temperature

**Figure 1:**
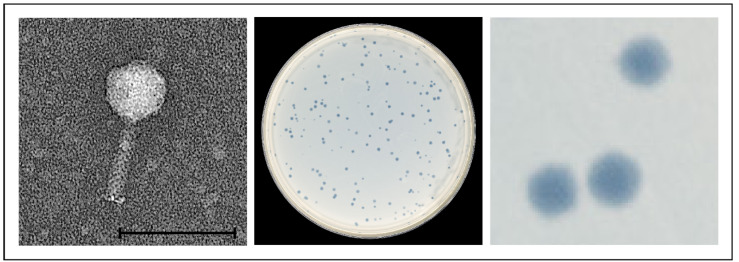
Morphology of and plaques produced by the temperate phage vB_YenM_29.18. The bar represents 100 nm.

To examine whether pathogenic *Y. enterocolitica* strains of our culture collection harbour active prophages, 102 arbitrarily selected strains belonging to B4/O:3 (n = 38), B2/O:5,27 (n = 26), B2/O:9 (n = 27) and 1B/O:8 (n = 11) were treated with mitomycin C. After incubation overnight, the optical density of the individual cultures differed significantly. Approximately half of the strains exhibited a decrease in optical density (A_600 nm_) by at least 0.5 compared to the control without mitomycin C and were thus regarded as potentially lysogenic. Lysates of the respective cultures were tested for lytic activity on all 102 strains at room temperature (RT), 28 °C and 37 °C. Nine lysates produced plaques on at least one indicator strain, but only at RT, not at 28 °C or 37 °C. Seven lysates originated from B2/O:5,27 strains, whereas one came from B2/O:9 and one from 1B/O:8 (Table 1). The plaques were tiny (<1 mm) and rather turbid (Figure 1); however, one strain (16-YE00006) produced two types of plaques, turbid and clear plaques representing two different phages. Upon three-fold isolation of single plaques, mass lysates were prepared from the phages, which then were analysed regarding their morphology and genome sequence.

### 2.2. The Phages Are Myoviruses and Belong to Three Distinct Groups

**Figure 2:**
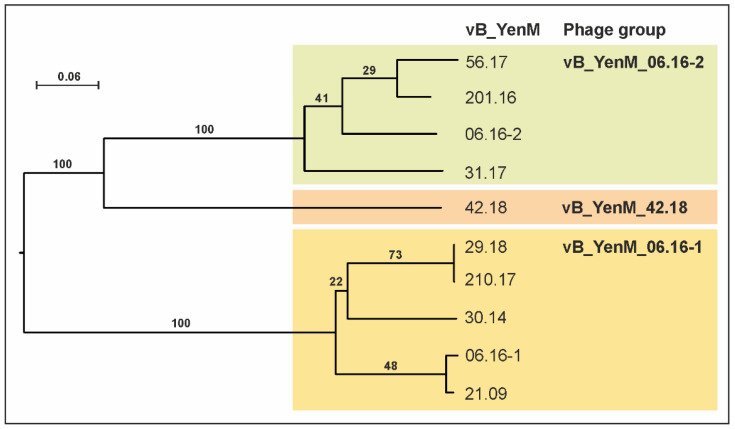
Phylogenomic GBDP tree of the temperate phages analysed in this study. Based on the phylogenomic GBDP tree of the phage genomes, seven species, two genera clusters and two family clusters were predicted. The numbers above branches are pseudo-bootstrap support values from 100 replications. The branch lengths are scaled in terms of the respective distance formula (formula D6; average support of 82%) used. The predicted branches are colored according to the assigned phage groups.

**Figure 3:**
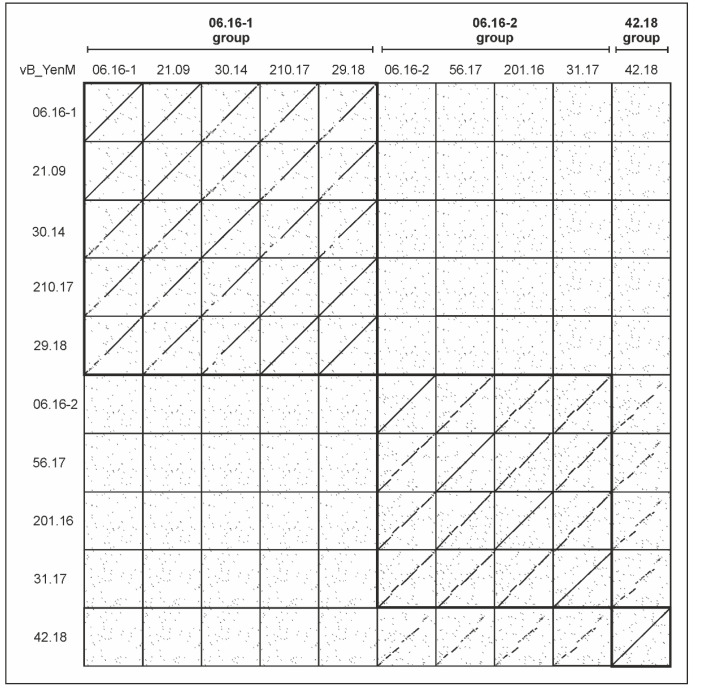
Multi-dot plot alignment of the phage genomes. Dot plot alignments of individual genome combinations were generated using DS Gene (v 2; Accelrys Inc., San Diego, CA, USA) using default parameters (window size 30, hash value 6, min. score 65) and composited to a multi-dot plot using Corel Draw X8 (V 18.1.0.690; Corel Corporation, Ottawa, ON, Canada).

Electron microscopy revealed that the ten phages possess a similar morphology with an isometric head and a contractile tail, typical for myoviruses (Figure 1, Supplemental Material Figure S1). However, genetically they are diverse and form three phylogenetic groups, of which group 3, represented by phage vB_YenM_42.18 is more closely related to group 2 than to group 1 (Figure 2 and Figure 3).

The first group consists of five phages with genome sizes between 43.8 and 44.9 kb. All of them were isolated from B2/O:5,27 strains. The type phage of this group is vB_YenM_06.16-1, to which the other members are closely related (70.7 to 97.4% identity, Figure 2 and Figure 3).

**Figure 4:**
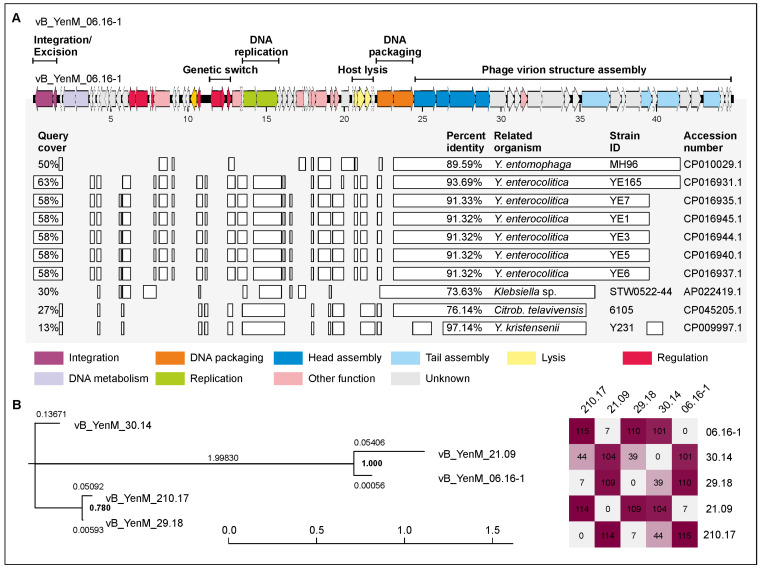
Gene map of vB_YenM_06.16-1 and its relationship to other prophages. (**A**) Gene map of the phage and identity values with other prophage sequences deposited in GenBank. White bars represent regions of high nucleotide similarity (>75%). (**B**) Phylogenetic tree (single nucleotide polymorphism (SNP)-based) of phages belonging to the vB_YenM_06.16-1 group. The scale bar represents the number of nucleotide substitutions per site. On the right, the numbers of single nucleotide variations between individual phage genomes are given. *Citrob*. *telavivensis* = *Citrobacter telavivensis*.

The genome of these phages is clearly divided into two arms, of which the right arm is more similar among the phages of this group than the left arm (Figure 4). While the left arm contains genes for an integrase and excisionase, DNA metabolism and replication, phage lifestyle repressors, host cell lysis proteins and terminase subunits, the right arm mainly comprises genes for virion assembly. At the nucleotide level, the phages show significant similarities to segments of putative prophages of *Y. enterocolitica* and *Y. entomophaga*, and, to a lesser extent, also to *Citrobacter* and *Klebsiella*, but not to any characterized phage. It is notable that particularly the right arm is very similar to prophage sequences, whereas homologous sequences in the left arm are rather scattered (Figure 4). However, it is yet not clear whether these sequences represent active prophages. Interestingly, using the PHASTER prophage prediction software, none of the phage genomes could be completely identified in the chromosome of their host strains; all of them were classified as incomplete or questionable (Supplemental Material Table S1). We also determined the attachment (*attP*) and integration (*int*) site of the phages. Even though *attP* is only seven nucleotides long, all phages of this group are surprisingly integrated at the same position within the bacterial chromosome, between genes coding for a pyridoxal phosphate-dependent aminotransferase and a methylthioribulose 1-phosphate dehydratase (Table 2).

**Figure 5:**
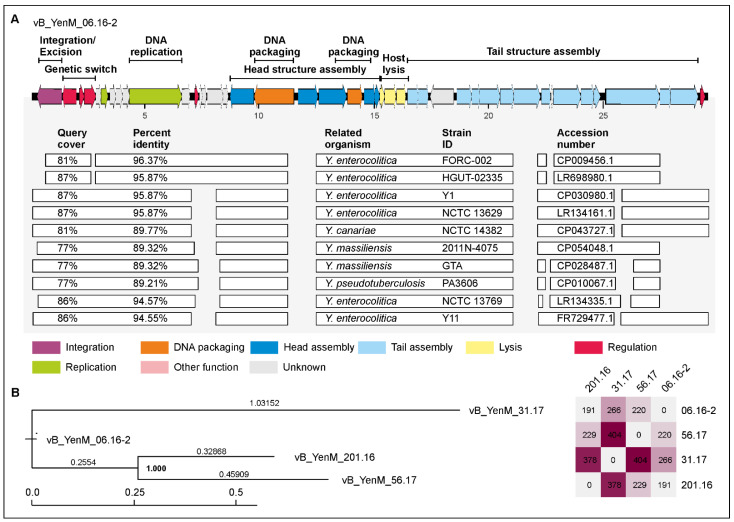
Gene map of vB_YenM_06.16-2 and its relationship to other prophages. (**A**) Gene map of the phage and identity values with other prophage sequences. White bars represent regions of high nucleotide similarity (>75%). (**B**) Phylogenetic tree (single nucleotide polymorphism (SNP)-based) of phages belonging to the vB_YenM_06.16-2 group. The scale bar represents the number of nucleotide substitutions per site. On the right, the number of single nucleotide variations between individual phage genomes are given.

The second group is composed of four phages, whose genomes are smaller (29.5 to 33.2 kb), but also closely related to each other (76.8 to 80.8% identical to the type phage vB_YenM_06.16-2) and to prophages identified in several *Yersinia* species (Figure 5). In contrast, no similarities exist to group 1 phages. A closer look at the gene map of vB_YenM_06.16-2 shows that the genes for head assembly and DNA packaging are not clustered as in group 1 and are located on both DNA strands (Figure 5).

Nevertheless, the alignment with chromosomal sequences of *Yersinia* strains deposited in the databases disclosed even higher overall identity values as with the phages of the other group. By PHASTER analysis, sequences of the prophages were identified, whose sizes are in good agreement with those of the sequenced phages. Moreover, two prophage sequences were classified as intact (Supplemental Material Table S1). Other striking differences between the groups relate to the *attP* and *int* sites. Unlike with group 1, *attP* of group 2 phages is much longer (50 and 57 nucleotides). While three group 2 phages possess almost identical *attP* sites, the site of vB_YenM_31.17 is completely different (Table 2). Therefore, it is not surprising that this phage is integrated at a different site (gene for a YcbX family protein) than the other members of this group (tRNA for isoleucin).

The third group currently contains only one phage (vB_YenM_42.18), which, however, was isolated from two B2/O:9 strains (data not shown) and which is also present in the *Y. enterocolitica* strain FDAARGOS_1090 (accession number CP068147.1, positions 1,036829 to 1,073307). This phage has a genome of 36,481 bp showing moderate sequence similarities to group 2 (Figure 3).

**Figure 6:**
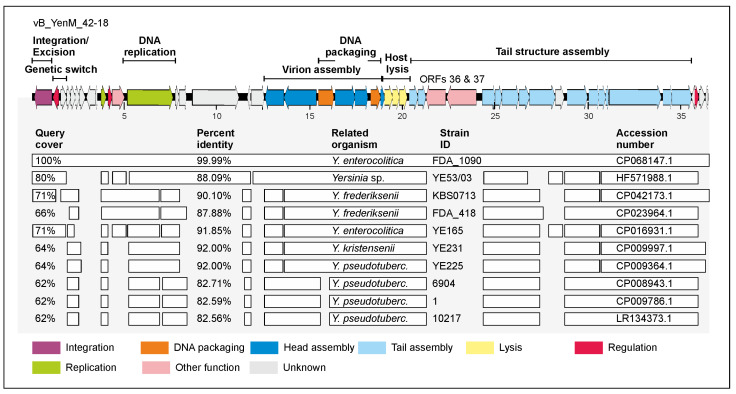
Gene map of phage vB_YenM_42.18 and its relationship to other prophages. Gene map of the phage and identity values with other prophage sequences. White bars represent regions of high nucleotide similarity (>75%).

In addition, the genome organization is similar to group 2 phages (Figure 5 and Figure 6). One striking difference to group 2 regards two genes encoding a fatty acid desaturase and a Acyl CoA oxidase that are present only in vB_YenM_42.18 (ORF36 and 37) and may be beneficial for the fatty acid metabolism of the hosts. Another difference pertains to the *attP* and *int* site of this phage. Unlike all other phages analysed in this study, *attP* of vB_YenM_42.18 is 11 nucleotides long and the phage is integrated in its host strain between genes for a metalloprotease PmbA and a ribosome-associated protein. (Table 2).

It is worth mentioning that genes probably associated with virulence or antibiotic resistance were not detected on any phage genome.

### 2.3. Group 2 Phages Have the Broadest Host Range

To determine the host specificity of the phages, 50 *Y. enterocolitica* strains, 30 strains belonging to other *Yersinia* species as well as various other genera of *Enterobacterales* (e.g., *Escherichia, Klebsiella*, *Salmonella* (Supplemental Material Table S2) were examined. All tests were performed at RT, 28 °C and also at 37 °C in the presence of CaCl_2_ and MgSO_4_, because these cations are sometimes important for phage-induced lysis of *Yersinia* strains [23]. However, in this study plaques were exclusively obtained at RT. With the exception of group 2 phage vB_YenM_56.17, which was able to lyse one *Y. frederiksenii* strain, all phages infected only *Y. enterocolitica*. However, the host specificity of the groups differed clearly. Group 1 phages showed the narrowest host range and infected almost exclusively B2/O:5,27 strains. The only exception was vB_YenM_06.16.1, which additionally lysed one B1A/O:5 strain (Table 3). By contrast, phages of group 2 and the group 3 phage vB_YenM_42.18 revealed an extended host range and additionally lysed both strains belonging to B2/O:5,27 and B4/O:3. Some phages additionally infected B2/O:9, 1B/O:8 and B1A strains of serotype O:5 and/or O:27 (Table 3). We then looked at the tail fiber proteins of the phages, generally important for host specificity. An alignment of these proteins disclosed almost identical tail fiber proteins of group 1, whereas the proteins of group 2 and 3 phages diverged in part significantly, particularly at the C-termini, which may explain the different host ranges (Supplemental Material Figures S2–S4).

## 3. Discussion

**Figure 7:**
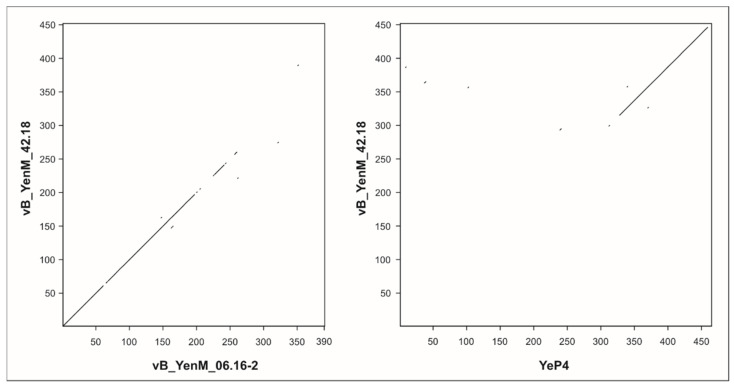
Relationship of the tail fiber protein of group 3 phage vB_YenM_42.18 to that of group 2 phage vB_YenM_06.16-2 and YeP4. Dot plot alignments were generated using DS Gene (v 2; Accelrys Inc., USA) using default parameters (window size 30, hash value 6, min. score 65) and composited to a multi-dot plot using Corel Draw X8 (V 18.1.0.690; Corel Corporation).

In this study, we have isolated and characterized ten temperate phages from pathogenic *Y. enterocolitica* strains. Since many prophage sequences were identified in our lysogenic strains by in silico analyses (Supplemental Material Table S1), it cannot be excluded that more prophages can be induced, perhaps by use of other inductors, such as UV or heat. All isolated phages are myoviruses, but they belong genetically to three novel groups. Most prophages were isolated from B2/O:5,27 strains, e.g., all group 1 phages. By contrast, phages of group 2 were isolated from strains of B2/O:5,27 and 1B/O:8, which may mean that group 2 prophages are more widespread in *Yersinia* than those of group 1. This hypothesis is corroborated by the fact that for group 2, much more similar prophage sequences are deposited in the databases than for group 1 and that the related sequences are much less segmented (Figure 4 and Figure 5). Moreover, group 2 phages showed a broader host range. Indeed, while phages of group 1 almost exclusively lysed B2/O:5,27 strains, all phages of group 2 infected also B4/O:3 strains; some of them additionally infected B2/O:9, 1B/O:8 and certain B1A strains suggesting that strains of several bio/serotypes can be lysogenized by group 2 phages. However, it is notable that none of the 38 tested B4/O:3 strains released a phage upon induction with mitomycin C. Moreover, interestingly, group 3 phage vB_YenM_42.18 isolated from B2/O:9, did not lyse any strain belonging to this bio/serotype (Table 3). The reason for this finding is as yet unclear. It cannot be ruled out that the tested strains contain a similar prophage or at least a prophage repressor silencing the lytic activity of the phage or restriction modification systems, which can cleave foreign DNA. We will examine these possibilities by bioinformatic analyses after sequencing of the B2/O:9 strains. Nevertheless, the heterogeneity of the group 2 phages’ host range is discernible by looking at the tail fiber proteins, which are much more diverse in this group than in group 1. An interesting observation was that the C-termini of the tail fiber proteins of the group 3 myovirus vB_YenM_42.18 and the podovirus YeP4 isolated in China [20] were almost identical, even though these two phages do not share any other similarity (Figure 7). Since both phages were isolated from serotype O:9, it is very likely that the tail fiber proteins play the leading role in host specificity of these phages. However, the fact that the group phages did not lyse the same B2/O:5,27 strains and that phage vB_YenM_06.16.1 was additionally able to infect a B1A/O:5 strain indicates that the host specificity may be influence by another yet not identified factor of the phages.

Group 3 phage vB_YenM_42.18 revealed a moderate relationship to group 2 and was similarly able to lyse strains belonging to different bio/serotypes. However, its genome is larger than that of group 2 phages and the genomic differences justify its allocation to an individual group. While active prophages of group 1 and 2 may coexist in the same cell, as it is the case with strain 16-YE00006, for vB_YenM_42.18 such a possibility is uncertain. The PHASTER analyses gave regrettably no answer to this question, because both phages of group 1 and 3 were not clearly predicted by this software, but blast alignments revealed that except for strain 18-YE00042, which harbors vB_YenM_42.18, none of the other eight strains contain a similar prophage.

Another difference between the three groups refers to the attachment and integration sites of the phages. For all group 1 phages, an identical *attP* sequence of only seven nucleotides was determined, even though the phages integrated at the same position within the *Y. enterocolitica* chromosome. Since this sequence exists in multiple copies in *Yersinia*, it is very likely that other sequences of the phages are important for site-specific integration. By contrast, *attP* of group 2 phages is much longer (50 to 57 nucleotides). This site probably only exists once in the *Yersinia* genome and thus, it is not surprising that three phages possessing the same or almost identical site integrate at the same position in a tRNA gene known to be a common site for phage integration. The exception is only vB_YenM_31.17, whose *attP* site is dissimilar to the others resulting in a divergent integration site. Comparison of the integrases of group 2 phages disclosed that the integrase of vB_YenM_31.17 differs significantly from those of the other group 2 phages (data not shown). Finally, group 3 phage vB_YenM_42.18 possesses a unique *attP* site of 11 nucleotides and integrates at a different position than the other phages.

Interestingly, all phages in this study form plaques only at room temperature (~22 °C). In this regard the temperate phages in this study resemble the virulent *Y. enterocolitica* phages phiR1-RT and vB_YenM_TG1 exhibiting lytic activity at and below 25 °C [24]. The host receptor for these two phages is OmpF, which is maximally expressed at 4 °C and strongly repressed at 37 °C. The fact that the prophages of this study can be induced at both room temperature and 28 °C, indicates that replication and virion assembly are not prevented at this temperature. However, the reason for the temperature-dependent lytic activity of these phages is obviously not the receptor, because phages containing a neomycin resistance gene were able to lysogenize the bacteria at 4 °C, 28 °C and even at 37 °C, which released the phages upon induction (data not shown). Popp et al. (2000) and Liang et al. (2019) performed their plaque assays at 28 °C and 25 °C, respectively [18,20]. While the phages in the former study clearly differed in this regard from those described here, it has regrettably not been reported whether the phages investigated in the latter publication are similarly unable to lyse their host strains at elevated temperatures.

In conclusion, this study showed that temperate phages can be isolated from various *Y. enterocolitica* bio/serotypes, but that specific groups of phages are apparently prevailing. No virulence or antibiotic resistance genes were identified on the phage genomes. To answer the question of whether these phages may contribute to horizontal gene transfer, transduction experiments will be carried out in the near future.

## 4. Materials and Methods

### 4.1. Bacterial Strains and Culture Conditions

All strains of this study originate from the culture collection of the Consiliary Laboratory for *Yersinia* (KL *Yersinia*) hosted at the German Federal Institute for Risk Assessment (BfR), Berlin, Germany. *Yersinia* spp. bacteria were cultivated in/on lysogeny broth (LB)-based media at 28 °C as previously described [23].

### 4.2. Isolation, Propagation and Purification of Phages

Phages described here were recovered from mitomycin C-treated *Y. enterocolitica* cultures (5 mL) as previously described [21,22]. After prophage induction, the lysates were subjected to centrifugation at 10,000*× g* for 20 min and 20 °C, followed by filtration of the supernatant through 0.22 µm pore-size filters (VWR International, Darmstadt, Germany). Lytic activity was detected by spotting 10 μL of serial dilutions of each lysate onto a lawn of *Y. enterocolitica* indicator strains belonging to various serotypes. Individual phages were purified by threefold recovery of single plaques. Mass lysates of the phages were obtained by preparing 10–20 agar plates with confluent lysis of the host bacteria. The soft agar was harvested by scraping and resuspended in SM-buffer for several hours. Thereafter, the lysates were centrifuged for 20 min at 10,000 × *g* to remove the agar and debris and then filtered (see above). Phages were concentrated by ultracentrifugation and used for whole-genome sequencing (WGS) and transmission electron microscopy (TEM).

### 4.3. Host Range Determination

For the preparation of softagar overlay plates, 100 to 200 μL of the respective indicator strain were mixed with 6 mL prewarmed NZCYM (VWR International, Darmstadt, Germany) soft agar (0.6%) and poured onto a LB agar plate [25]. Lytic activity was determined by spot assays as described above. After incubation overnight at RT, 28 °C and 37 °C, plaques were visually inspected and counted [23].

### 4.4. Transmission Electron Microscopy

Purified phages were subjected to negative staining with uranyl acetate (VWR International, Darmstadt, Germany) as previously described [26]. TEM was conducted using a JEM-1010 (JEOL, Tokyo, Japan) at 80 kV acceleration voltage [23].

### 4.5. Phage DNA Preparation, Short-Read WGS and Bioinformatics Analysis

WGS was conducted using phage DNA extracted from purified virions as previously described (23). DNA sequencing libraries were prepared using the Nextera Flex DNA Sample Preparation Kit and subjected to short-read sequencing (2 × 151 cycles) on an NextSeq 500 (Illumina, San Diego, CA, USA) [27]. Raw read trimming and *de novo* genome assembly was conducted with the Aquamis pipeline [28]. Illumina sequencing resulted in single contigs representing the respective genome sizes by exhibiting a sequencing depth of at least 250. Manual curation was conducted to remove duplicated sequences and to adjust the genome sequences according to its organization in the prophage state. Prediction of coding sequences (CDS) was performed with the annotation tool of PATRIC (www.patricbrc.org; accessed in 15 November 2021) [29]. Transcription terminators and transfer RNAs were predicted using ARNold (http://rssf.i2bc.paris-saclay.fr/toolbox/arnold/; access date: 29 January 2022) and tRNAscan-SE 1.21 (http://lowelab.ucsc.edu/tRNAscan-SE/; access date: 29 January 2022), respectively. For the detection of genes involved in resistance development against antimicrobials, biocides and heavy metals or factors associated with virulence/pathogenicity of bacteria, the Virulence Factor Database (VFDB; http://www.mgc.ac.cn/VFs/; accessed in 15 November 2021) as well as the databases of ResFinder 4.1 (https://cge.cbs.dtu.dk/services/ResFinder/; accessed in 15 November 2021) and CARD (https://card.mcmaster.ca/; accessed in 15 November 2021) were screened against the phage genomes using MyDbFinder 2.0 (default settings; https://cge.cbs.dtu.dk/services/MyDbFinder/; accessed in 15 November 2021).

### 4.6. Comparative Phage Genome Analyses

Pairwise comparison of our sequenced phage genomes was conducted using the Genome-BLAST Distance Phylogeny (GBDP) method for prokaryotic viruses [30]. Intergenomic distances were used for the generation of a balanced minimum evolution tree. Taxon boundaries were estimated using the OPTSIL program as specified [31]. SNP-based phylogenetic analysis was performed using the CSI Phylogeny tool 1.4 (https://cge.cbs.dtu.dk/services/CSIPhylogeny/; accessed in 1 February 2022) of the Center for Genomic Epidemiology [32]. For aligning DNA or amino acid sequences, Clustal Omega (https://www.ebi.ac.uk/Tools/msa/clustalo/; accessed in 1 February 2022) was used under default settings. Dot plot analysis was conducted using the software DS Gene (v2.5, Accelrys Inc., San Diego, CA, USA) as specified (standard settings: window size 30, hash value 6, min. score 65). By using the blastn suite of NCBI for comparison of sequenced *Y. enterocolitica* phages to potential prophage sequences, the search was conducted using the standard database (nucleotide collection (nr/nt)) optimized for highly similar sequences (megablast).

### 4.7. Nucleotide Sequencing Data

The nucleotide sequences of the phages were deposited in GenBank under the accession numbers: OM046622 (vB_YenM_06.16-1), OM046621 (vB_YenM_29.18), OM046623 (vB_YenM_21.09), OM046624 (vB_YenM_42.18), OM046625 (vB_YenM_210.17), OM046626 (vB_YenM_06.16-2), OM046627 (vB_YenM_56.17), OM046628 (vB_YenM_201.16), OM046629 (vB_YenM_30.14) and OM140653 (vB_YenM_31.17). The genomes of the *Y. enterocolitica* host isolates are accessible under bioproject PRJNA789480 (individual accession numbers: 14-YE00030, JAJTNN000000000; 16-YE00006, JAJTNM000000000; 16-YE00201, JAJTNL000000000; 17-YE00031, JAJTNK000000000; 17-YE00056, JAJTNJ000000000; 17-YE00210, JAJTNI000000000; 18-YE00029, JAJTNH000000000; 18-YE00042, JAJTNO000000000 and 21-YE00009, JAJTNG000000000).

## Figures and Tables

**Table 1:** Lysogenic *Y. enterocolitica* strains and released phages.

Strain (Accession No.)	Bio/ Serotype	Source (Year, German Federal State)	Phage (Accession No.)
14-YE00030 (JAJTNN000000000)	B2/O:5,27	Pork (2014, Saxony)	vB_YenM_30.14 (OM046629)
16-YE00006 (JAJTNM000000000)	B2/O:5,27	Pork (2016, Saxony Anhalt)	vB_YenM_06.16-1 (OM046622), vB_YenM_06-16-2 (OM046626)
16-YE00201 (JAJTNL000000000)	1B/O:8	Wildlife (2016, Berlin)	vB_YenM_201.16 (OM046628)
17-YE00031 (JAJTNK000000000)	B2/O:5,27	Pork (2017, Saxony)	vB_YenM_31.17 (OM140653)
17-YE00056 (JAJTNJ000000000)	B2/O:5,27	Pork (2017, Saxony Anhalt)	vB_YenM_56.17 (OM046627)
17-YE00210 (JAJTNI000000000)	B2/O:5,27	Sheep feces (2017, Schleswig-Holstein)	vB_YenM_210.17 (OM046625)
18-YE00029 (JAJTNH000000000)	B2/O:5,27	Pork (2018, Saxony)	vB_YenM_29.18 (OM046621)
18-YE00042 (JAJTNO000000000)	B2/O:9	Pork (2018, Saxony)	vB_YenM_42.18 (OM046624)
09-YE00021 (JAJTNG000000000)	B2/O:5,27	Pork (2009, Saxony Anhalt)	vB_YenM_21.09 (OM046623)

**Table 2:** Attachment sites of the groups vB_YenM_06-16-1, vB_YenM_06-16-2 and vB_YenM_42-18.

vB_YenM Phage	Nucleotide Sequence of the Attachment Site (Size in Nucleotides) 5′-3′ Direction	*Y. enterocolitica* Host	Integration Site (Affected Element)
**vB_YenM_06.16-1 group**			
06.16-1	ATAACAC (7 nt)	16-YE00006	Intergenic ^A^
21.09	ATAACAC (7 nt)	21-YE00009	Intergenic ^A^
29.18	ATAACAC (7 nt)	18-YE00029	Intergenic ^A^
30.14	ATAACAC (7 nt)	14-YE00030	Intergenic ^A^
210.17	ATAACAC (7 nt)	17-YE00210	Intergenic ^A^
**vB_YenM_06.16-2 group**			
06.16-2	GACTCATAATCGCTTGGTCACTGGTTCAAGTCCAGTAGGGGCCACCAAATTTTAGCT (57 nt)	16-YE00006	tRNA-Ile
31.17	AAAATCCCTCGGCTTATGGCTGTGCGGGTTCAAGTCCCGCCCCGGGCACCATGGAAA (57 nt)	17-YE00031	YcbX family protein
56.17	GACTCATAATCGCTTGGTCACTGGTTCAAGTCCAGTAGGGGCCACCAAAT (50 nt)	17-YE00056	tRNA-Ile
201.16	GACTCATAATCGCTTGGTCACTGGTTCAAGTCCAGTAGGGGCCACCAAAT (50 nt)	16-YE00201	tRNA-Ile
**vB_YenM_42.18 group**			
42.18	ACAACCTGCTA (11 nt)	18-YE00042	Intergenic ^B^

^A^ intergenic sequence between genes coding for the pyridoxal phosphate-dependent aminotransferase and the methylthioribulose 1-phosphate dehydratase. ^B^ intergenic sequence between genes coding for a metalloprotease and a ribosome-associated protein.

**Table 3:** Host range of the phages on *Y. enterocolitica* isolates at room temperature (RT).

Phage Groups	1					2				3
Isolate ID	06.16-1	21.09	29.18	30.14	210.17	06.16-2	31.17	56.17	201.16	42.18
1A (O-type)										
13-YE00003 (O:8)	-	-	-	-	-	-	-	-	-	-
12-YE00021 (O:7,8)	-	-	-	-	-	-	-	-	-	-
14-YE00065 (O:5)	-	-	-	-	-	-	-	+	-	-
18-YE00017 (O:5)	+	-	-	-	-	+	-	+	-	-
13-YE00025 (O:5,27)	-	-	-	-	-	-	+	-	-	-
16-YE00080 (O:27)	-	-	-	-	-	-	-	+	-	-
16-YE00083 (O:27)	-	-	-	-	-	-	-	-	-	-
13-YE00019 (O:3)	-	-	-	-	-	-	-	-	-	-
12-YE00030 (O:6,31)	-	-	-	-	-	-	-	-	-	-
07-YE00015 (O:13,17)	-	-	-	-	-	-	-	-	-	-
17-YE00192 (O:41,43)	-	-	-	-	-	-	-	-	-	-
11-YE00037 (rough)	-	-	-	-	-	-	-	-	-	-
11-YE00038 (rough)	-	-	-	-	-	-	-	-	-	-
13-YE00020 (n.d.)	-	-	-	-	-	-	-	-	-	-
**B1/O:8**										
YE181 * (DSM 27689)	-	-	-	-	-	-	-	-	-	-
13-YE00006	-	-	-	-	-	-	-	+	+	-
16-YE00128	-	-	-	-	-	-	-	-	-	-
16-YE00196	-	-	-	-	-	-	-	+	+	-
16-YE00197	-	-	-	-	-	-	-	+	+	-
16-YE00198	-	-	-	-	-	-	-	+	+	-
16-YE00201	-	-	-	-	-	-	-	-	-	-
17-YE00142	-	-	-	-	-	-	-	-	-	-
**B2/O:9**										
YE182 *	-	-	-	-	-	-	-	-	-	-
YE183 * (Evira 663)	-	-	-	-	-	-	-	-	-	-
16-YE00235	-	-	-	-	-	-	-	-	-	-
17-YE00133	-	-	-	-	-	-	-	-	-	-
17-YE00134	-	-	-	-	-	-	-	-	-	-
17-YE00143	-	-	-	-	-	-	-	-	-	-
17-YE00155	-	-	-	-	-	-	-	-	-	-
17-YE00218	-	-	-	-	-	-	-	+	-	-
18-YE00008	-	-	-	-	-	-	-	+	+	-
18-YE00014	-	-	-	-	-	-	-	-	-	-
18-YE00022	-	-	-	-	-	+	-	+	+	-
**B2/O:5,27**										
YE180 * (DSM 11504)	+	+	-	-	-	-	+	+	+	-
17-YE00021	+	+	+	+	+	+	+	+	+	-
17-YE00031	+	+	+	+	+	+	-	+	+	-
17-YE00041	+	+	-	+	-	+	-	+	+	-
17-YE00056	-	-	-	-	-	+	+	-	+	+
17-YE00097	+	+	-	-	+	+	+	+	+	-
17-YE00169	-	-	-	-	+	-	-	-	+	-
17-YE00203	+	+	+	+	+	+	+	+	+	-
**B4/O:3**										
YE179 * (DSM 9676)	-	-	-	-	-	+	-	+	-	+
YE184 * (Evira 595)	-	-	-	-	-	+	-	-	-	-
17-YE00038	-	-	-	-	-	-	-	-	-	-
17-YE00058	-	-	-	-	-	-	+	-	+	-
17-YE00168	-	-	-	-	-	+	-	+	+	+
17-YE00222	-	-	-	-	-	+	-	+	+	+
18-YE00001	-	-	-	-	-	+	-	+	+	+
18-YE00010	-	-	-	-	-	+	-	+	+	+
18-YE00025	-	-	-	-	-	+	-	+	+	+

Type phages of the groups 1, 2 and 3 are indicated in bold. Symbols: +, plaque formation detectable; -, no plaque formation.

## Data Availability

Not applicable.

## Supplementary Materials

The following supporting information can be downloaded at: www.mdpi.com/article/10.3390/ijms23126779/s1.
