# Supplementary material for: Host Range, Morphology and Sequence Analysis of Ten Temperate Phages Isolated from Pathogenic Yersinia enterocolitica Strains
Source: Int J Mol Sci. 2022 Jun 17;23(12):6779. doi: 10.3390/ijms23126779 (PMC9224151; doi:10.3390/ijms23126779)
Supplement: Supplementary file 1 [file ijms-23-06779-s001.zip › ijms-1757941-supplementary.pdf]

Supplemental Material

Host range, morphology and sequence analysis of ten temperate phages isolated from pathogenic *Yersinia enterocolitica* strains

Jens Andre Hammerl [1], Sabrin El-Mustapha [1], Michelle Bölcke [1], Hannah Trampert [1], Andrea Barac [1], Claudia Jäckel [1], Ashish K. Gadicherla [1] and Stefan Hertwig [1,*]

[1] *German Federal Institute for Risk Assessment, Department of Biological Safety, Max-Dohrn Str. 8-10, D-10589 Berlin, Germany*; (J.A.H.: jens-andre.hammerl@bfr.bund.de, S.El-M.: sabrin-elmustapha95@outlook.de, M.B.: michelleboelcke@web.de, H.T.: hannah.trampert@web.de, A.B.: andrea.barac@bfr.bund.de, C.J.: claudia.jaeckel@bfr.bund.de, A.G.: ashish.gadicherla@bfr.bund.de and S.H.: stefan.hertwig@bfr.bund.de)

* Correspondence: stefan.hertwig@bfr.bund.de; Tel.: +49 30 18412 24502

Supplemental Material Figures

Figure S1. Transmission electron micrographs of temperate *Y. enterocolitica* phages. The bar represents 50 nm.

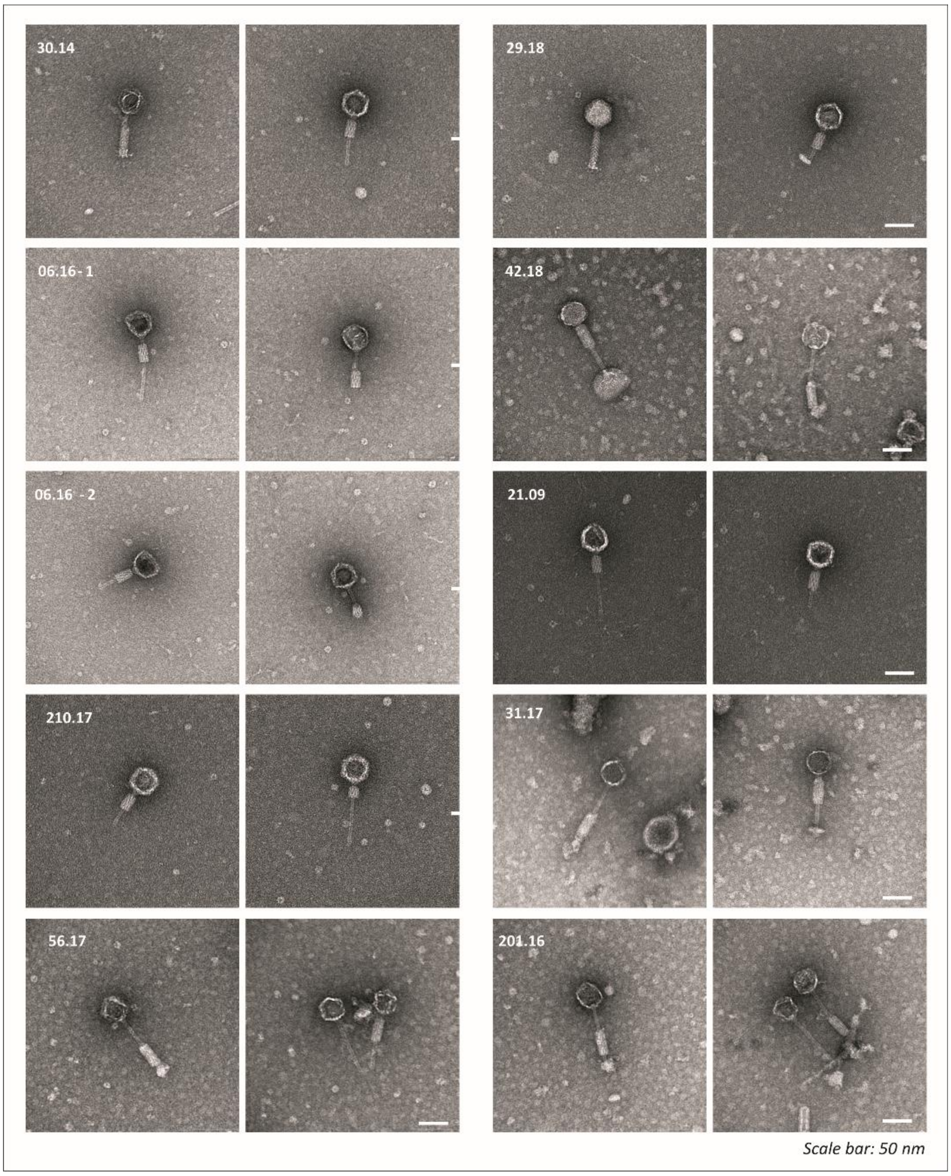

Figure S2. Comparison of the tail fiber protein sequences of *Y. enterocolitica* group 1 phages. (A) Amino acid alignment of the protein sequences, (B) phylogenetic relationship of the sequences using CSIphylogeny (default parameters) and (C) percentage of relationship between the individual protein sequences.

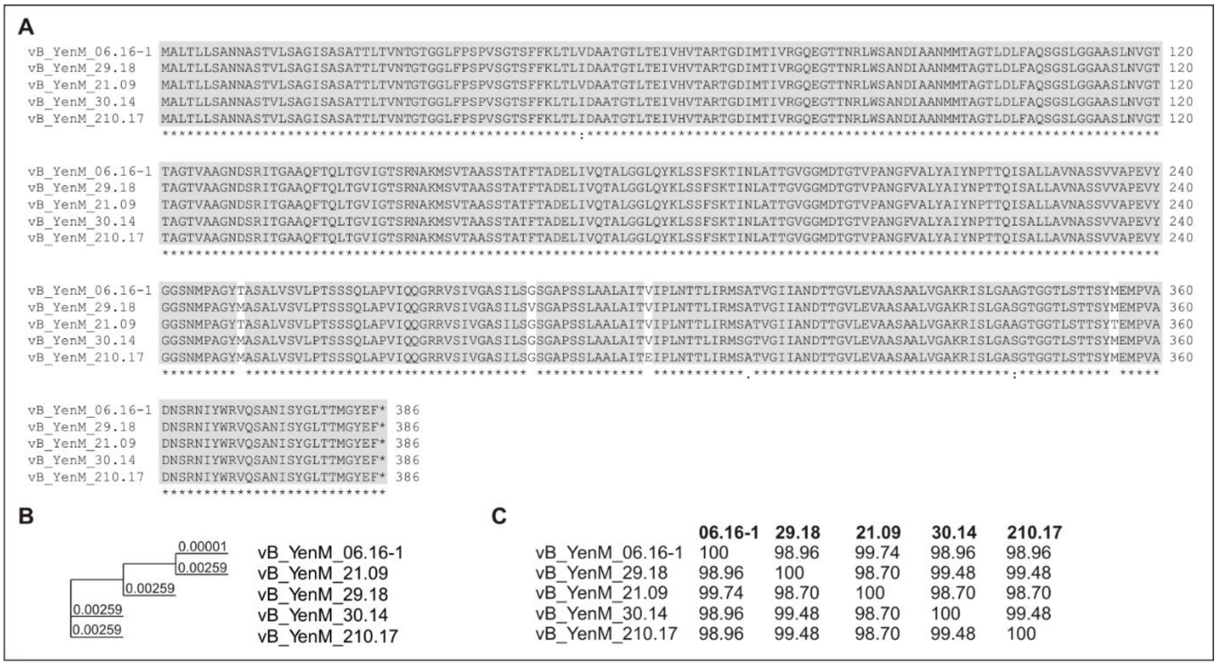

Figure S3. Comparison of the tail fiber protein sequences of *Y. enterocolitica* group 2 phages. (A) Amino acid alignment of the protein sequences, (B) phylogenetic relationship of the sequences using CSIphylogeny (default parameters) and (C) percentage of relationship between the individual protein sequences.

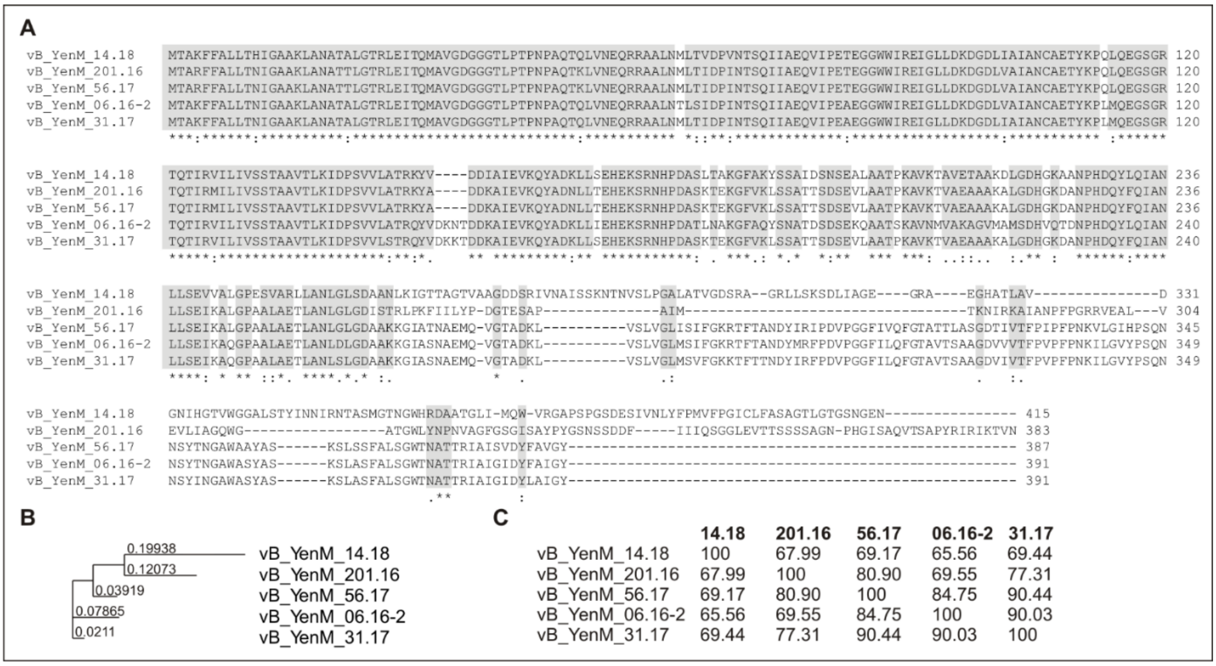

Figure S4. Amino acid alignment of the tail fiber protein sequences of *Y. enterocolitica* group 3 phage vB_YenM_42.18 and YeP4.

```
YEP4          MAVPNQTPYTVYTANGVTTVFPFNFMV-FSATDLAVSINGTVLSSGFTVSGVGIVNGGAVTFLTPPASGVTVMLARVMPLVRVT-EYQDNGDLLAATVNKDFDRIWMVLQGQAVDHS-LA 117
vB_YenM_42.18 --------------------MTVKYFVLLTNLGAAKLANAAALGTQLQITQMAVGDGGGALPTPNPAQTQLIAEKRRAALNSLSIDEANSSQIIAEQVIPETDGGWWIREVGLFDKDGIL 100
                                    : .:::* ::  . *   *.:.*.: : :: :.: :**..     **.   :   *   *  :: :  :..:::*  *  : *  * : :   .*:. :

YEP4          LSRPSI--AYD--YYAGRGYR----ITN----------------------IAAPINDQDATTKSYVDALAHSNDQHTLRVPESVVGVIPSVALRSNKLLGFSDSGEPVAIDAASGSAAD 206
vB_YenM_42.18 IAIANCPDTYKPQLQEGSGRTQTVRMVLIVSSTEAITLKIDPSVVLATRQYVDKKVDDKAIEVKQYADKLLADHEKSR-N--------HPDASLTAK---GFAKYSSA------TTSDSE 202
              ::  .   :*.      * *      :.                      :   ::* :  .*.*.* *  .:::    .          *..:* ::   **:. ..       : * ::

YEP4          VLIKLAKPTGADLVGYSTGTVKSGLDNAISIINTNIQLPGNLGVKGLEVHP----------------MGSNPTE--GGQIDLYDKADARAAFIDIDSGGNFRVVTESLGVTLSINRTTGN 308
vB_YenM_42.18 VLAA--TP----------KAVKTAVETA----------AKDLGDHGKAANPHDQYLQIANLLSEVVALGPESVARLLANLGLSDAA-----NLKIGTTAGTVAAGDDSRIVNAIQSTNTA 295
              **    .*          :**:.::.*          :** :*   .:*                :* :  .     .::.* * *      :.*.: ..   .. :.   :. :*: *.

YEP4          IVIPKALTAAGILKGYGIIAEGTVYAGNTAAWLAADGNVYGSVWGGYLSTYLSNRTEHRVRAWAAVQGNGTIISSFGFAAINRTNVGGYNFTMSTSNGAYAVTVGINGGSQNGALNAHSA 428
vB_YenM_42.18 ISLPGSLTTAGTVTGGGISSVGSVYAGNTAAWLAADGNVYGSVWGGYLSTYLSNRTEHRVRAWAAVQGNGTIISSFGFSAINRTNVGGYNFTMSTSNGAYAVTVGINGGSQNGALNAHSA 415
              * :* :**:** :.* ** : *:*******************************************************:*****************************************

YEP4          NIWNRTPNSFSIQNANDSGTQYNWIDWPEFYVIVVGP 465
vB_YenM_42.18 NIWNRTPNSFSIQNANDSGTQYNWIDWPEFYVIVVGP 452
              *************************************
```

Supplemental Material Tables

Table S1. Phage and prophage prediction using PHASTER

Y. enterocolitica **phage/isolate**	**PHASTER result** (size, status, score, related phage, accession number, G+C content)	**Position**
vB_YenM_30.14	28.1 kb, incomplete, 60, *Salmonella* 118970_sal3, NC_031940, 46.88%	49-28,246 (C1)
14-YE00030	41.1 kb, intact, 150, *Salmonella* 118970_sal3, NC_031940, 46.43%	40,333-81,441 (C18)
	24.9 kb, intact, 130, *Erwinia* ENT90, NC_019932, 48.35%	1-24,937 (C50)
	31 kb, intact, 120, *Vibrio* 12B12, NC_021070, 47.94%	143,495-174,510 (C4)
	56.6 kb, questionable, 70, *Salmonella* 118970_sal3, NC_031940, 47.84%	12,699-69,397 (C6)
	26.7 kb, incomplete, 60, Enterobacteria PsP3, NC_005340, 45.49%	121-26,826 (C28)
	12 kb, incomplete, 50, *Aeromonas* phiO18P, NC_009542, 42.76	74,291-86,291 (C7)
	15.1 kb, incomplete, 40, *Escherichia* 500465_1, NC_049342, 46.56%	2-15,132 (C15)
	9.8 kb, incomplete, 40, *Salmonella* SEN34, NC_028699, 41.26%	32-9,875 (C40)
vB_YenM_06.16-1	28.3 kb, questionable, 80, Enterobacteria ST104, NC_005841, 47.76%	49-28,422 (C1)
vB_YenM_06.16-2	28.9 kb, intact, 150, *Erwinia* ENT90, NC_019932, 50.81%	539-29,475 (C1)
16-YE00006	40.8 kb, intact, 150, *Salmonella* 118970_sal3, NC_031940, 46.44%	40,333-81,195 (C20)
	31 kb, intact, 120, *Vibrio* 12B12, NC_021070, 47.94%	37,061-68,076 (C1)
	45.3 kb, questionable, 90, Enterobacteria ST104, NC_005841, 48.43%	19,265-64,644 (C7)
	38.6 kb, questionable, 70, *Erwinia* ENT90, NC_019932, 50.84%	882-39,511 (C40)
	26.7 kb, incomplete, 60, Enterobacteria PsP3, NC_005340, 45.49%	242,995-269,700 (C1)
	12 kb, incomplete, 50, *Aeromonas* phiO18P, NC_009542, 42.77%	74,405-86,404 (C17)
	9.8 kb, incomplete, 50, Enterobacteria mEp237, NC_019704, 41.28%	24,183-34,018 (C41)
	15.1 kb, incomplete, 40, *Escherichia* 500465_1, NC_049342, 46.56%	2-15,132 (C16)
	14.9 kb, incomplete, 10, Enterobacteria 186, NC_001317, 41.97%	269-15,258 (C22)
vB_YenM_201.16	29.6 kb, incomplete, 60, *Erwinia* ENT90, NC_019932, 51.51%	519-30,211 (C1)
16-YE00201	7.9 kb, questionable, 90, Enterobacteria phiP27, NC_003356, 45.01%	4,278-12,189 (C56)
	35.6 kb, questionable, 80, *Erwinia* ENT90, NC_019932, 47.84%	778-36,424 (C43)
	36 kb, questionable, 70, *Erwinia* ENT90, NC_019932, 50.92%	116,217-152,217 (C3)
	7.7 kb, questionable, 70, *Escherichia* 500465_1, NC_049342, 47.62%	45,787-53,530 (C28)
	11.9 kb, incomplete, 40, Enterobacteria P4, NC_001609, 43.44%	46-12,013 (C47)
	24.8 kb, incomplete, 30, *Escherichia* 500465_1, NC_049342, 38.22%	10,034-34,912 (C47)

vB_YenM_31.17	32.7 kb, intact, 150, *Erwinia* ENT90, NC_019932, 48.95%	
17-YE00031	23.4 kb, intact, 150, *Erwinia* ENT90, NC_019932, 52.07%	42,366-65,853 (C26)
	17.8 kb, intact, 150, *Erwinia* ENT90, NC_019932, 51.35%	450-18,249 (C54)
	31 kb, intact, 120, *Vibrio* 12B12, NC_021070, 47.94%	01,887-232,902 (C3)
	26.7 kb, incomplete, 60, Enterobacteria PsP3, NC_005340, 45.49%	252-26,957 (C3)
	9.8 kb, incomplete, 50, Enterobacteria mEp237, NC_019704, 41.26%	24,202-34,053 (C40)
	12 kb, incomplete, 50, *Aeromonas* phiO18P, NC_009542, 42.76%	74,390-86,390 (C5)
	22.2 kb, incomplete, 40, *Escherichia* 500465_1, NC_049342, 46.07%	74,715-96,966 (C14)
	14 kb, incomplete, 10, Enterobacteria 186, NC_001317, 41.55%	65,769-79,814 (C19)
vB_YenM_56.17	31.1 kb, incomplete, 60, *Erwinia* ENT90, NC_019932, 50.72%	32-32,750 (C1)
17-YE00056	40.2 kb, intact, 150, Salmonella 118970_sal3, NC_031940, 46.45%	16,476-56,750 (C30)
	31 kb, intact, 120, *Vibrio* 12B12, NC_021070, 47.94%	37,077-68,092 (C4)
	24 kb, intact, 120, *Erwinia* ENT90, NC_019932, 48.14%	2-24,069 (C49)
	26.8 kb, questionable, 70, Enterobacteria PsP3, NC_005340, 45.47%	1-26,883 (C29)
	33.2 kb, incomplete, 60, *Erwinia* ENT90, NC_019932, 49.71%	360-33,605 (C16)
	12 kb, incomplete, 50, *Cronobacter* ESSI_2, NC_047854, 42.79%	71,938-83,968 (C6)
	9.9 kb, incomplete, 50, Enterobacteria mEp237, NC_019704, 41.16%	24,202-34,117 (C39)
	22.2 kb, incomplete, 40, *Escherichia* 500465_1, NC_049342, 46.07%	74,685-96,888 (C18)
vB_YenM_210.17	27.3 kb, incomplete, 60, Enterobacteria 933W, NC_000924, 47.73%	49-27,368 (C1)
17-YE00210	40.2 kb, intact, 150, *Salmonella* 118970_sal3, NC_031940, 46.44%	40,349-80,596 (C20)
	31 kb, intact, 120, *Vibrio* 12B12, NC_021070, 47.94%	20,887-232,902 (C2)
	31 kb, questionable, 70, Enterobacteria 933W, NC_000924, 48.02%	100,198-131,209 (C6)
	26.7 kb, incomplete, 60, Enterobacteria PsP3, NC_005340, 45.49%	252-26,957 (C2)
	12 kb, incomplete, 50, *Aeromonas* phiO18P, NC_009542, 42.79%	71,938-83,968 (C5)
	9.8 kb, incomplete, 50, Enterobacteria mEp237, NC_019704, 41.28%	24,202-34,037 (C38)
	22.2 kb, incomplete, 40, *Escherichia* 500465_1, NC_049342, 46.07%	74,703-96,906 (C16)
	15.2 kb, incomplete, 10, Enterobacteria 186, NC_001317, 42.22%	1-15,274 (C21)
vB_YenM_29.18	27.3 kb, incomplete, 60, Enterobacteria 933W, NC_000924, 47.73%	49-27,368 (C1)
18-YE00029	31 kb, intact, 120, *Vibrio* 12B12, NC_021070, 47.94%	117,722-148,737 (C4)
	36.6 kb, intact, 100, *Vibrio* VBM1, NC_020850, 47.01%	138,390-175,167 (C5)
	26.7 kb, incomplete, 60, Enterobacteria PsP3, NC_005340, 45.49%	101-26,806 (C28)
	12 kb, incomplete, 50, *Cronobacter* ESSI_2, NC_047854, 42.79%	71,902-83,932 (C7)
	9.8 kb, incomplete, 50, *Salmonella* SEN34, NC_028699, 41.25%	24,163-34,039 (C40)

	18.1 kb, incomplete, 30, *Salmonella* 118970_sal3, NC_031940, 48.06%	29,219-47,393 (C33)
	12 kb, incomplete, 30, *Escherichia* 500465_1, NC_049342, 46.28%	84,374-96,436 (C16)
	15.2 kb, incomplete, 10, Enterobacteria 186, NC_001317, 42.20%	64,796-80,045 (C21)
vB_YenM_42.18	11.2 kb, incomplete, 10, *Klebsiella* 4LV2017, NC_047818, 51.27%	904-12,183 (C1)
	8.7 kb, incomplete, 30, *Klebsiella* OXA48phi14.1, NC_049454, 50.84%	15,228-23,954 (C1)
	7.1 kb, incomplete, 20, *Klebsiella* OXA245phi4.2, NC_049449, 46.69%	28,164-35,325 (C1)
18-YE00042	65.1 kb, intact, 150, Enterobacteria mEp390, NC_019721, 45.20%	84,251-149,443 (C5)
	13.6 kb, incomplete, 60, Enterobacteria PsP3, NC_005340, 44.34%	11,458-25,093 (C51)
	7.2 kb, incomplete, 50, *Salmonella* SEN34, NC_028699, 43.65%	38,110-45,358 (C34)
	28.9 kb, incomplete, 30, *Salmonella* Fels_2, NC_010463, 45.80%	121,090-150,004 (C7)
	7.3 kb, incomplete, 30, *Erwinia* ENT90, NC_019932, 49.97%	2-7,316 (C71)
	10.6 kb, incomplete, 10, Enterobacteria 186, NC_001317, 40.67%	12,714-23,363 (C52)
vB_YenM_21.09	28.2 kb, questionable, 80, *Salmonella* SE1, NC_011802, 47.93%	49-28,303 (C1)
21-YE00009	31 kb, intact, 120, *Vibrio* 12B12, NC_021070, 47.94%	37,029-68,004 (C2)
	11.6 kb, intact, 110, *Salmonella* 118970_sal3, NC_031940, 50.99%	533-12,182 (C60)
	28.2 kb, questionable, 90, *Salmonella* 118970_sal3, NC_031940, 44.52%	305-28,581 (C24)
	31.9 kb, questionable, 90, *Salmonella* SE1, NC_011802, 48.18%	25,706-57,652 (C25)
	26.7 kb, incomplete, 60, Enterobacteria PsP3, NC_005340, 45.49%	242,963-269,668 (C2)
	12 kb, incomplete, 50, *Cronobacter* ESSI_2, NC_047854, 42.76%	74,186-86,186 (C6)
	12.1 kb, incomplete, 30, *Escherichia* 500465_1, NC_049342, 46.30%	311-12,430 (C15)
	15.2 kb, incomplete, 10, Enterobacteria 186, NC_001317, 42.19%	64,784-80,021 (C19)

In grey, predicted prophage matching to the respective phage genomes are indicated. Additionally to the nucleotide positions within the WGS sequences of the bacteria, further information about the respective contig is given in brackets.

Table S2. ***Enterobacterales*** **isolates tested for lytic activity**

**Phage group**	**1**					**2**				**3**
**Isolate ID**	**06.16-1**	09.21	29.18	30.14	210.17	**06.16-2**	31.17	56.17	201.16	**42.18**
***Klebsiella* spp. (n=10)**										
K. pneumoniae (n=2)	-	-	-	-	-	-	-	-	-	-
K. oxytoca (n=2)	-	-	-	-	-	-	-	-	-	-
K. variicola (n=2)	-	-	-	-	-	-	-	-	-	-
K. michiganensis (n=2)	-	-	-	-	-	-	-	-	-	-
K. quasipneum. (n=2)	-	-	-	-	-	-	-	-	-	-
***Escherichia* spp. (n=10)**										
E. coli (O157, n=2)	-	-	-	-	-	-	-	-	-	-
E. coli (O104, n=2)	-	-	-	-	-	-	-	-	-	-
E. coli (O26, n=2)	-	-	-	-	-	-	-	-	-	-
E. hermannii (n=2)	-	-	-	-	-	-	-	-	-	-
E. marmotae (n=2)	-	-	-	-	-	-	-	-	-	-
***Salmonella* serovar (n=10)**										
S. Typhimurium (n=2)	-	-	-	-	-	-	-	-	-	-
S. Braenderup (n=2)	-	-	-	-	-	-	-	-	-	-
S. Schwarzengrund (n=2)	-	-	-	-	-	-	-	-	-	-
S. Enteritidis (n=2)	-	-	-	-	-	-	-	-	-	-
S. Paratyphi (n=2)	-	-	-	-	-	-	-	-	-	-
**Other bacteria (n=10)**										
Proteus mirabilis (n=2)	-	-	-	-	-	-	-	-	-	-
Enterobacter cloacae (n=2)	-	-	-	-	-	-	-	-	-	-
Morganella morganii (n=2)	-	-	-	-	-	-	-	-	-	-
Citrobacter freundii (n=2)	-	-	-	-	-	-	-	-	-	-
Raoultella ornitholytica (n=2)	-	-	-	-	-	-	-	-	-	-

Type phages of the respective groups are indicated bold.
